# Mn–Co/MIL-100(Fe) nanocomposite for pseudocapacitive energy storage and adsorptive removal of malachite green dye

**DOI:** 10.1039/d6ra07914h

**Published:** 2026-11-04

**Authors:** Ayesha Bibi, Arfaa Sajid, Qaisar Manzoor, Ajwa Jabeen, Muhammad Hassan, Faiza Imtiaz, Awais Ahmad, Muhammad Iftikhar Khan

**Affiliations:** a Department of Chemistry, The University of Lahore Lahore Pakistan arfaa.sajid@chem.uol.edu.pk awaisahmed@gcuf.edu.pk; b Faculty of Engineering and Technology, Shinawatra University Sam Khok Thailand; c Department of Physics, The University of Lahore Lahore Pakistan; d Department of Physics, Faculty of Science, Islamic University of Madinah Madinah 42351 Saudi Arabia

## Abstract

A Mn–Co bimetallic nanoparticle-doped MIL-100(Fe) composite (Mn–Co/MIL-100(Fe)) was synthesized by solvothermal synthesis and evaluated for supercapacitor and dye-adsorption applications, with pristine MIL-100(Fe) used for direct comparison. The optical analysis showed a 1.85 eV band gap, indicating electronic interactions between the bimetallic species and the MOF matrix. Morphological studies confirmed the formation of hetero-interfaces, showing a transition from the spherical morphology of pristine MIL-100 to irregular polyhedral forms coated with Mn–Co nanoparticles. Additionally, Brunauer–Emmett–Teller (BET) analysis revealed a microporous structure having 17.71 m^2^ g^−1^ surface area, providing accessible pathways for electrolyte-ion diffusion. Cyclic voltammetry (CV) analysis revealed pseudo-capacitive behavior linked to faradaic redox reactions at the Mn and Co active sites, with power-law (*b*-value) and Dunn's method analyses showing a pronounced shift from diffusion-controlled charge storage in pristine MIL-100(Fe) (*b* = 0.45) to predominantly surface-controlled, capacitive-dominated behavior in the Mn–Co/MIL-100(Fe) nanocomposite (*b* = 0.99, up to ∼84% capacitive contribution). GCD measurements showed a specific capacitance of 167.9 F g^−1^ at 0.8 A g^−1^ for the Mn–Co/MIL-100(Fe) nanocomposite, approximately 2.5-fold higher than pristine MIL-100(Fe) (66.2 F g^−1^) under the same conditions. Cycling stability over 52 charge–discharge cycles showed no capacitance fading, with specific capacitance and coulombic efficiency both increasing to ∼170 F g^−1^ and ∼65.6%, respectively, indicative of progressive electrochemical activation. EIS analysis found a 31.87 Ω charge-transfer resistance, demonstrating moderate ion transport and charge-transfer properties within the porous framework. Beyond its pseudocapacitive performance, the Mn–Co/MIL-100(Fe) nanocomposite achieved ∼95% adsorptive removal efficiency toward malachite green dye, following pseudo-second-order kinetics (*R*^2^ = 0.9998) indicative of a chemisorption mechanism. These results indicate that Mn–Co incorporation modifies the MIL-100(Fe) properties, enhancing its potential for advanced multifunctional energy storage and water-remediation applications.

## Abstract 2

Doping of Mn–Co bimetallic nanoparticles into the MIL-100(Fe) framework to form a hetero-interfaced Mn–Co/MIL-100(Fe) composite for pseudocapacitive energy storage.

## Graphical abstract

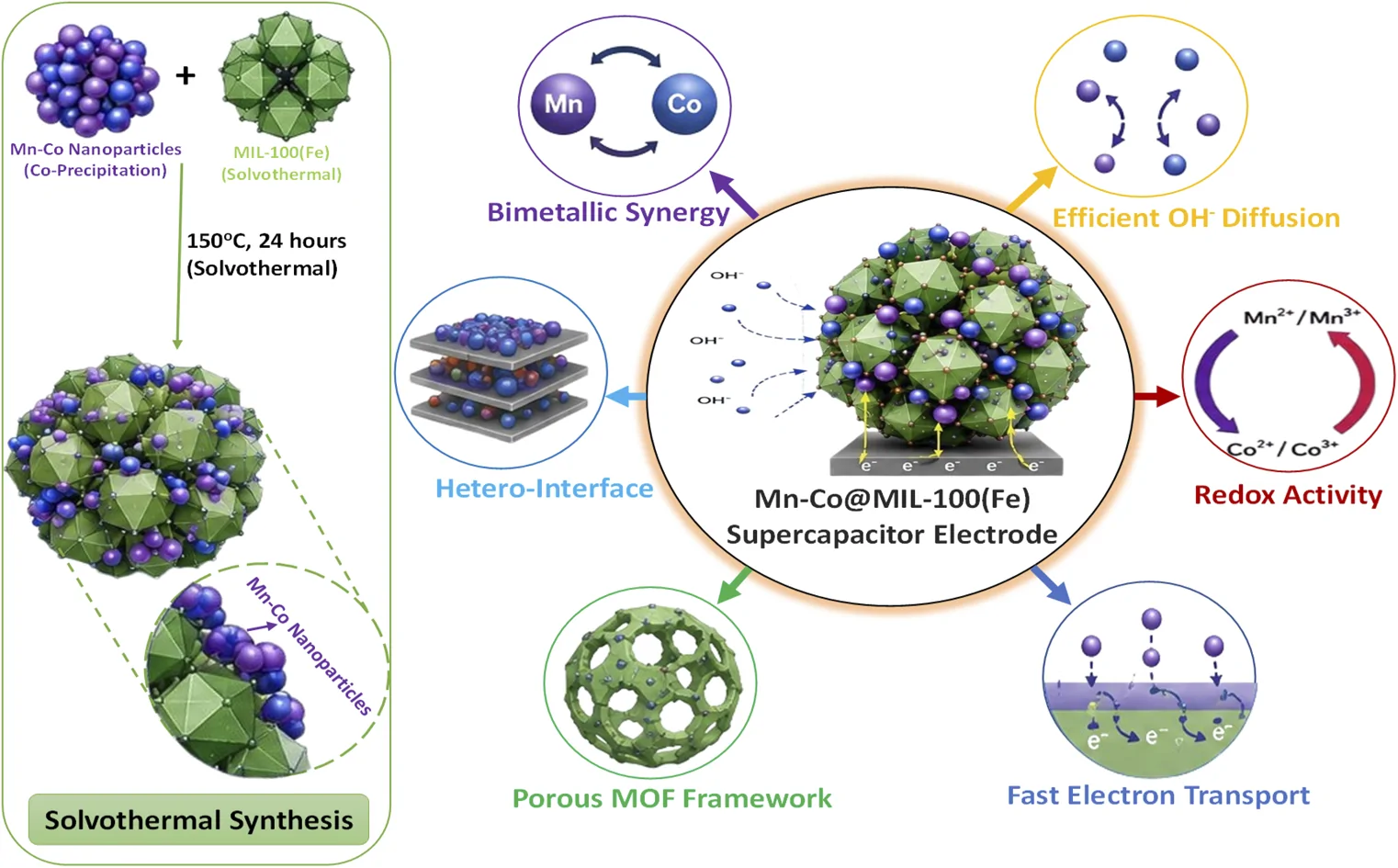

## 1 Introduction

Conventional energy resources like coal, natural gas and oil have been the key power source for the growing global population, industrialization, and technology. However, excessive consumption of these nonrenewable resources has created important environmental issues, including pollution, greenhouse gas emissions, and global warming. Transportation energy and electrical energy are in high demand among various forms of energy.^1^ This has pushed research and policy attention toward solar, wind, and hydroelectric power. These renewable sources have expanded quickly and now supply a meaningful share of the global electricity, yet a persistent problem remains: their output fluctuates with weather and time of day, making it difficult to guarantee a steady, reliable supply. For renewable energy to be deployed at scale and to sincerely support a sustainable grid, storage systems capable of banking surplus energy during peak generation and releasing it when demand rises are essential.^2,3^

The increasing number of electric vehicles, smart grids, and portable electronic devices has placed new and often conflicting demands on energy storage technology. Devices are now expected to deliver high power density, fast charging and discharging, and long operational lifetimes, all without sacrificing reliability. Conventional rechargeable batteries, while capable of storing large amounts of energy, tend to fall short on power density and charge–discharge speed. This gap has motivated the development of storage technologies designed specifically to address these deficiencies.^4,5^

Supercapacitors have emerged as a particularly promising option in this landscape because they strike a balance between the high energy density of batteries and the rapid charge–discharge behavior of conventional capacitors. Their combination of high power density, fast cycling, and long cycle life makes them well suited for applications ranging from portable electronics and hybrid electric vehicles to backup power and renewable-energy integration.^6,7^ Supercapacitors store energy *via* electrostatic charge accumulation in electric double layer capacitors (EDLCs) and reversible faradaic redox reactions in pseudocapacitors. Hybrid supercapacitors combine the advantages of both mechanisms, offering improved energy and power density.^8,9^

The properties of the electrode materials, which affect charge storage, conductivity, surface area, and electrochemical stability, have a significant impact on supercapacitor performance. Carbon-based electrodes, such as activated carbon, graphene, and carbon nanotubes, possess large surface area and conductivity but are limited in their energy density by relatively low values.^10,11^ Even though most metal oxides are not electrically conductive and could slow down the electrochemical action, transition metal hydroxides and oxides, including MnO_2_, Co_3_O_4_, NiO, and RuO_2_, provide a superior pseudo-capacitance due to reversible redox potential.^12^ Fast redox activity is offered by conducting polymers, *e.g.*, polyaniline, polypyrrole, and PEDOT; however, they tend to be mechanically unstable during long-term cycling.^13,14^

MOFs are emerging as suitable electrode materials with a highly porous structure, tunable metal centers, and large surface area to enable electrolyte ion diffusion and provide redox-active sites in large numbers. MIL-series MOFs, such as MIL-100, are particularly desirable because of their chemical stability, 3D networks of porosity, and potential for electrochemical application. MIL-100 is a porous solid with high surface area, in which ions can be transported efficiently, and metal clusters present in the framework provide possible redox-active sites that may facilitate the pseudo-capacitive reactions.^15,16^

Nevertheless, pure MIL-100 has a low electrical conductivity similar to most MOFs, and therefore it cannot be utilized as an electrode in high-performance supercapacitors.^17–19^ These limitations can be overcome by the incorporation of conductive materials, the formation of MOF-derived nanostructures, and the integration of multiple metal centers to form bimetallic structures. Such methods can enhance the capacitance and cycling stability, electrochemically active sites, and charge transfer.^20–22^

In particular, bimetallic transition metal compounds, such as manganese-cobalt systems, have attracted significant interest as advanced electrode materials. Compared with single-metal analogues, the bimetallic systems have synergistic interactions between different metal ions, which result in increased electrical conductivity, redox-active sites, and fast transfer of charge during electrochemical reactions. Manganese is relatively inexpensive, abundant, and environmentally friendly;^23^ however, its multiple oxidation states can provide additional redox-active sites, so its low conductivity limits its performance when used alone. Cobalt-based materials exhibit favorable redox kinetics and excellent pseudocapacitive behavior. So, the Mn–Co bimetallic structure demonstrates potential to be used in next-generation supercapacitor applications, as they provide a synergistic effect that improves all aspects of electrochemical activity.^24–26^

Malachite green (MG), a triphenylmethane cationic dye widely used in the textile, leather, and paper industries, shows serious environmental and health concerns due to its high solubility, chemical stability, and resistance to biodegradation. MG has been widely reported to exhibit toxicological concerns, including mutagenic, carcinogenic, teratogenic, and cytotoxic effects on aquatic organisms and mammalian systems, even at relatively low exposure concentrations.^27–29^ Conventional treatment methods such as coagulation, biological degradation, and chemical oxidation are often limited by high operational costs, incomplete mineralization, and generation of toxic byproducts. In this context, adsorption has emerged as a simple, cost-effective, and highly efficient technique for dye removal, with porous adsorbents offering large surface area and abundant active sites for pollutant capture.^30,31^ Consequently, the development of multifunctional, high-surface-area materials capable of simultaneously addressing energy storage and water remediation challenges has become an important research direction, motivating the present investigation into the dual applicability of Mn–Co/MIL-100(Fe) nanocomposite.

Most existing literature on MIL-100(Fe) composites relies on single-metal doping or physical mixing with conductive additives, which limits the synergistic benefits achievable through bimetallic incorporation. While a few studies have explored bimetallic MIL-100(Fe) frameworks, notably as supports for noble-metal catalysts in hydrogenation reactions, these efforts have been confined to catalytic applications, and no prior report has evaluated a Mn–Co bimetallic MIL-100(Fe) composite for dual functionality spanning both electrochemical energy storage and environmental remediation. This study addresses this gap by reporting the doping of Mn–Co bimetallic nanoparticles into the MIL-100(Fe) framework to form a hetero-interfaced Mn–Co/MIL-100(Fe) composite, whose structural, morphological, optical, electrochemical performance, and removal efficacy toward MG dye were systematically characterized, thereby establishing its potential as a multifunctional material for integrated energy-storage and water-treatment applications.

## 2 Experimental

### 2.1 Materials and reagents

Manganese(ii) acetate tetrahydrate (CH_3_COO)_2_Mn·4H_2_O, >98.0%, Kosdaq, China, cobalt(ii) chloride hexahydrate (CoCl_2_·6H_2_O, 99.0%, UNI-chem), NaOH (97%, Chemart, Tianjin, China), poly ethylene glycol (PEG, Macklin, China), nitric acid (HNO_3_, 65–68%, Huaweiruike, Beijing, China), *N*,*N*-dimethylformamide (DMF), ethanol (95%, Macklin, Shanghai, China) and iron(iii) chloride hexahydrate (FeCl_3_·6H_2_O, ≥97%), trimesic acid (H_3_BTC, 1,3,5 benzenetricarboxylic acid, 95%) were supplied by Sigma-Aldrich. Throughout the experiment, the water source was deionized water.

### 2.2 Synthesis of Mn–Co bimetallic nanoparticles

Co-precipitation method was employed for preparation of Mn–Co bimetallic nanoparticles with minor modifications to a previously reported procedure.^32^ 0.49 g of (CH_3_COO)_2_ Mn·4H_2_O was mixed in 50 mL of water, followed by the addition of 0.475 g of CoCl_2_·6H_2_O, and the mixture was stirred on hot plate until homogeneous. Subsequently, 0.1 g of polyethylene glycol (PEG) was added as a capping agent and mixed until fully dissolved by keeping the pH of soln. 9–10. The mixture was stirred continuously until a self-stable brownish colored precursor solution was obtained. The solution was centrifuged for 10 minutes at 5000 rpm to obtain the precipitate. Three volumes of distilled water were then added to it to eliminate remaining ions and contaminants. The synthesis of the Mn–Co bimetallic nanoparticle precursor was established by drying the purified precipitate of the sample in the oven at 60–80 °C that yielded a blackish powder. The dried product was then calcined at 360 °C to make Mn–Co bimetallic nanoparticles for 5 hours.

### 2.3 Synthesis of MIL-100(Fe) by solvothermal method

After some minor modifications, MIL-100(Fe) was produced using previously described methods.^33^ The solvothermal synthesis process involved self-assembly of FeCl_3_·6H_2_O and trimesic acid (H_3_BTC) in an autoclave to yield the metal–organic framework. Solution A (metal salt solution) was generally prepared by adding 1.62 g of FeCl_3_·6H_2_O to 20 mL of ethanol and stirring the mixture on a heated plate under constant magnetic agitation. As an acid modulator, two drops of concentrated HNO_3_ were added to prevent the hydrolysis of the metal precursor and boost the crystallization of MIL-100(Fe). The mixture was stirred until it became clear and homogeneous. Solution B (linker solution) was prepared by adding 1.26 g of trimesic acid in 20 mL of DMF; after that, the solution was sonicated for 20 minutes for complete dissolution and the linker solution was added dropwise under constant stirring. The mixture was further sonicated for 15–30 minutes to ensure a well-homogenized solution was obtained and transferred to autoclave coated for reaction at 170 °C for 24 hours. The desired product was obtained by centrifugation at 5000 rpm. The purified product was dried at 60–80 °C to form an orange powder, which was centrifuged twice with distilled water and twice with ethanol (MIL-100 Fe).

### 2.4 Fabrication of Mn–Co@MIL-100(Fe)

Mn–Co@MIL-100(Fe) synthesis was done by a modified solvothermal method and is schematically presented in Fig. 1. Mn–Co bimetallic nanoparticles were dispersed on a heated plate with 0.1 g of material initially suspended in 5 mL of ethanol until a uniformly dispersed blackish suspension was formed. The solution was sonicated for 20–30 minutes at 60 °C. In order to allow good homogenization, the suspension of nanoparticles was dropwise added into a transparent yellow metal salt solution prepared by mixing 1.22 g of FeCl_3_·6H_2_O in 10 mL of ethanol under continuous stirring. In the case of the linker solution, the metal salt-nanoparticle mixture was constantly stirred as 0.95 g of trimesic acid (H_3_BTC) was dropwise added once dissolved in 15 mL of dimethylformamide. The solution obtained after 30 minutes of sonication was put in an autoclave and heated at 150 °C for 24 hours. The product was centrifuged after the reaction and washed twice with ethanol and distilled water, followed by drying the product in a Petri dish at 80 °C. The synthesized Mn–Co@MIL-100(Fe) was converted to a dark brown powder as shown in Fig. 1.^33^

**Fig. 1:**
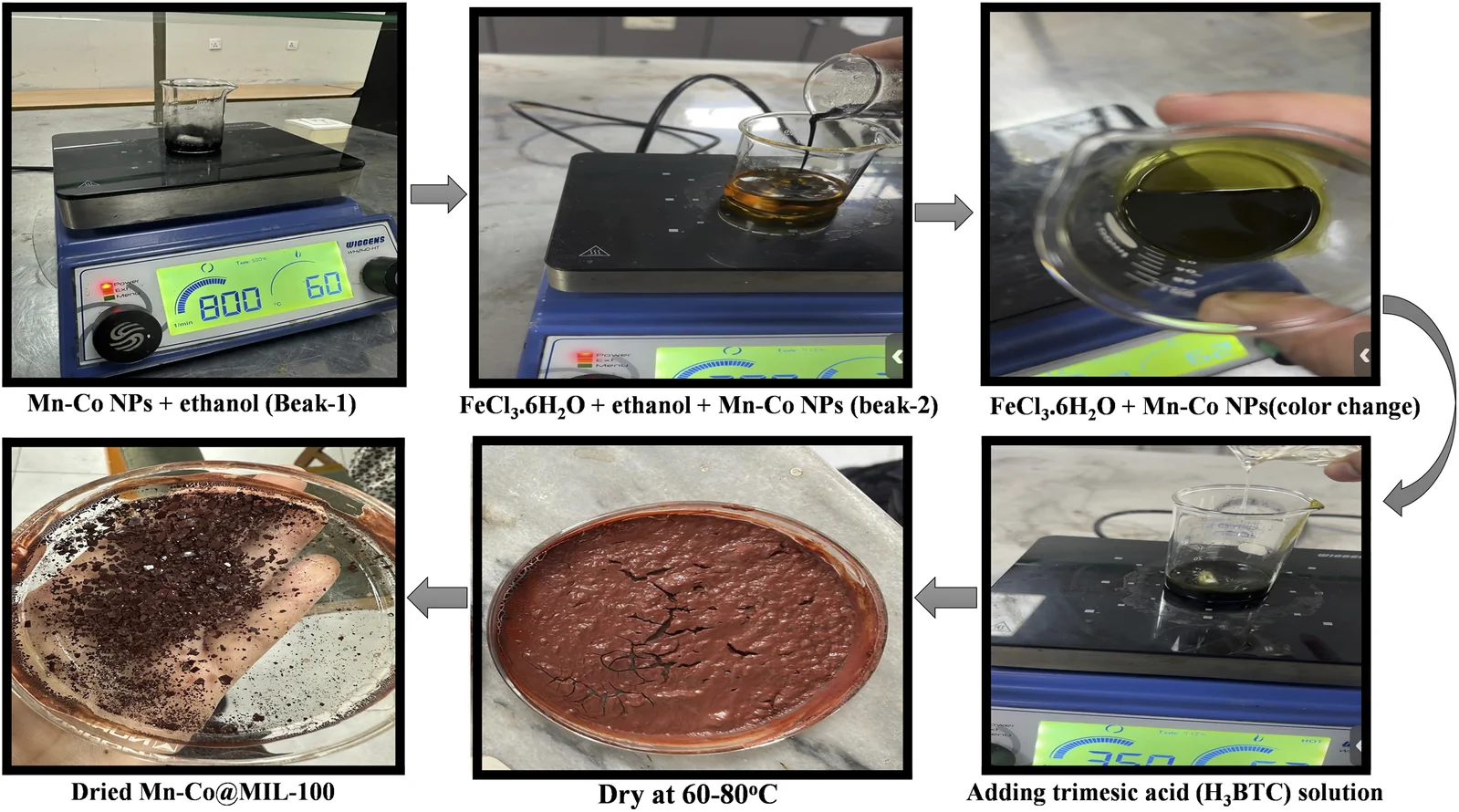
Photographic representation of Mn–Co@MIL-100 framework *via* a modified solvothermal method.

### 2.5 Electrode preparation

Before electrode fabrication, the nickel foam was pretreated to remove surface impurities and improve adhesion of the active material. A piece of nickel foam was dried at 60–70 °C in an oven over 5–6 hours after it had been placed in a 1 : 3 mixture of nitric acid and distilled water, and this was placed in an oven after five minutes. Mn–Co@MIL-100 was taken as the active material to make the electrode. Typically, polyvinylidene fluoride (PVDF) was incorporated as a binder, and 80 mg of MOF was mixed with 10 mg of carbon black to enhance electrical conductivity. A homogeneous slurry of 8 : 1 : 1 weight ratio (active material, carbon black, and binder) was formed through careful blending of the mixture. The active loaded mass was estimated by taking the difference between the end and starting weight of the nickel foam where the slurry was loaded on the prepared foam, and the mass of the dry slurry was dried in the oven at 80 °C for 24 hours.^14^

### 2.6 Material characterization

The sample was characterized to examine its structural, morphological, textural, and optical properties. The crystallinity and phase purity were studied by the use of XRD over a 2*θ* range of 5–80°. Functional groups and bonding interactions in the 500–4000 cm^−1^ region were investigated through FT-IR. Surface shape and distribution of particle size were studied with the help of SEM. The BET method was employed to measure specific surface area. Optical features and the band gap energy in the wavelength range of 200–800 nm were evaluated with the help of the Tauc plot method and UV-visible spectroscopy. These combined techniques confirmed the successful synthesis of Mn–Co bimetallic nanoparticles, MIL-100, and the Mn–Co@MIL-100 composite.

### 2.7 Electrochemical characterization

The electrochemical performance of the synthesized pristine MIL-100(Fe) and Mn–Co/MIL-100(Fe) electrodes was evaluated using CV, galvanostatic charge–discharge (GCD), and electrochemical impedance spectroscopy (EIS) in a three-electrode configuration on a CorrTest electrochemical workstation, with the synthesized material serving as the working electrode, Ag/AgCl as the reference electrode, and a platinum wire as the counter electrode. All measurements were carried out in an aqueous 1 M KOH electrolyte. CV measurements were conducted at scan rates of 5–50 mV s^−1^ over a potential window of 0–0.8 V for both electrodes, to evaluate redox activity and to determine the charge-storage mechanism (diffusion-*vs.* surface-controlled contributions) *via* power-law (*b*-value) and Dunn's method analyses. GCD measurements were performed at current densities of 0.8–2 A g^−1^ over a potential window of 0–0.6 V for pristine MIL-100(Fe) and 0–0.5 V for the Mn–Co/MIL-100(Fe) nanocomposite, to determine specific capacitance, energy density, power density, and cycling stability. EIS measurements were carried out over a frequency range of 100 kHz–0.01 Hz to determine solution resistance (*R*_s_), charge-transfer resistance (*R*_ct_), and ion-diffusion characteristics. Together, these complementary techniques provided a comprehensive understanding of the capacitive behavior, conductivity, and electrochemical dynamics of both electrode materials under identical measurement conditions.

### 2.8 Malachite green (MG) adsorption study

Removal of MG dye from aqueous solution by MIL-100(Fe) and Mn–Co/MIL-100(Fe) nanocomposites was investigated using the batch adsorption method. A stock solution of MG dye (20 ppm) was prepared in deionized water, and 20 mL of this solution was used for each adsorption experiment. The adsorbent dosage was fixed at 1 g L^−1^, and the mixture was maintained at room temperature throughout the experiment. The pH of the MG dye solution was measured, and all adsorption experiments were performed at natural, unadjusted solution pH without further modification. To evaluate the adsorption kinetics, aliquots were withdrawn at predetermined time intervals (10, 30, 45, 60, 120, and 180 min), followed by centrifugation to remove the adsorbent particles. The residual MG concentration in the supernatant was determined using a UV-vis spectrophotometer by monitoring the characteristic absorbance at *λ*_max_ = 615 nm. The percentage removal (%*R*) of MG dye was calculated using eqn (1):1
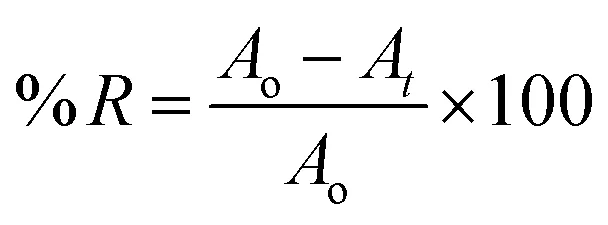
where *A*_o_ and *A*_*t*_ represent the absorbance of the MG dye solution before and after adsorption, respectively.

## 3 Results and discussion

### 3.1 UV-vis spectroscopy

UV-vis absorption spectra are shown in Fig. 2, where (a) represents Mn–Co nanoparticles, (b) MIL-100(Fe), and (c) shows the Mn–Co@MIL-100 composite. The Mn–Co bimetallic nanoparticles exhibited semiconducting behavior, with an absorption edge extending into the visible range due to electronic transitions between the conduction and valence bands. The optical band gap was estimated using the Tauc eqn (2):^34^

2 (*αhν*)^2^ ∼ (*hν* − *E*_g_)

where *α* is the absorption coefficient, *h* is Planck's constant, *v* is the frequency of light, and *E*_g_ is the energy of the optical band gap. It was discovered that Mn–Co nanoparticles possessed a band gap of 2.86 eV.^35^

**Fig. 2:**
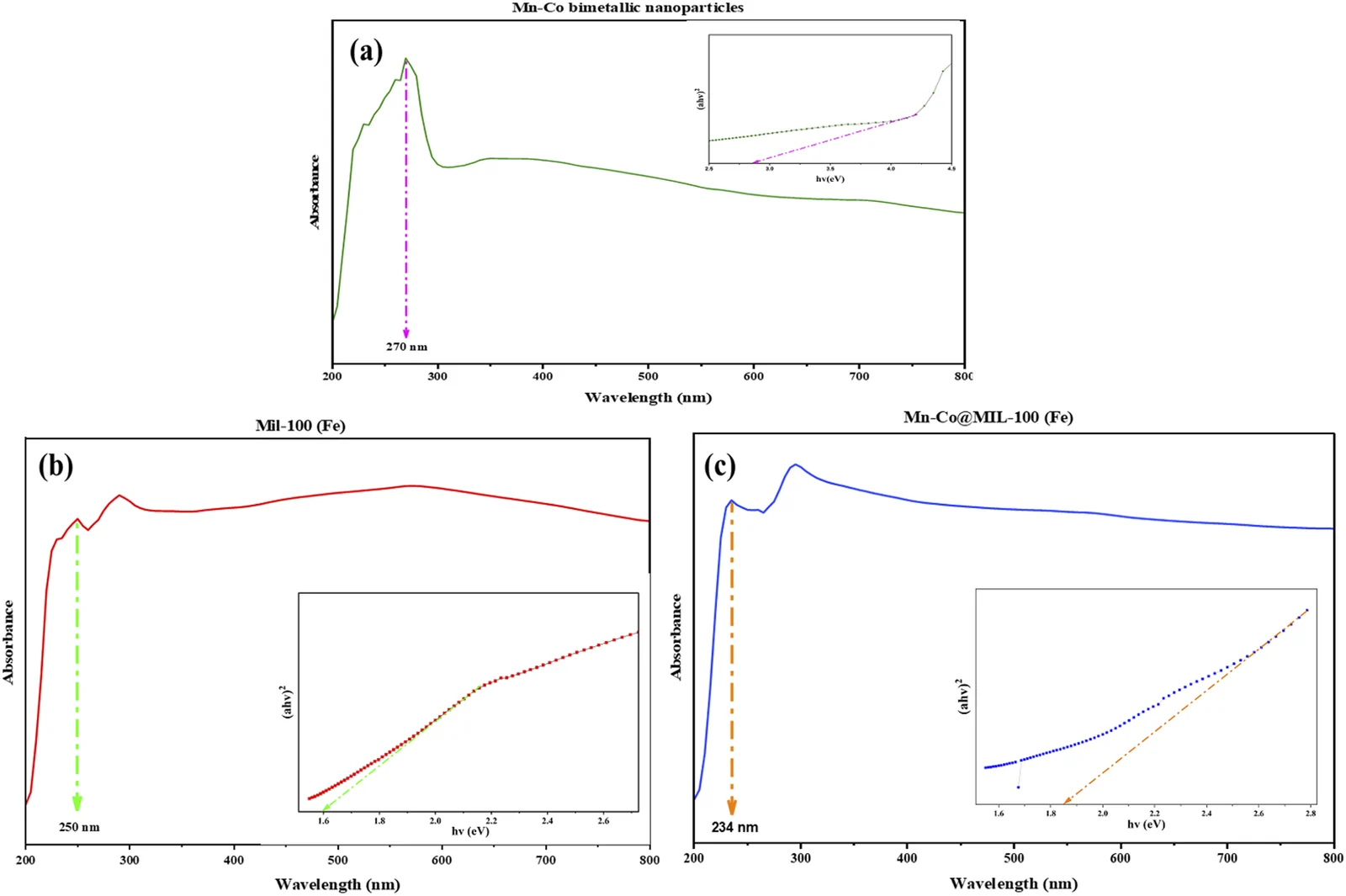
UV-vis absorption spectra and corresponding Tauc plots of (a) Mn–Co NPs, (b) MIL-100 (Fe), and (c) Mn–Co@MIL-100 composite.

MIL-100(Fe) spectrum in Fig. 2b showed a characteristic absorption band at approximately 240 nm, which is attributed to ligand-to-metal charge transfer (LMCT) transitions between the organic linker and the metal centers.^36,37^ The same Tauc relation was used to obtain the band gap of 1.6 eV, which lies within the range of frequencies reported as 1.6 to 2.7 eV.^21,38–41^ The absorption features of MIL-100 were mainly maintained in the Mn–Co@MIL-100 composite, which implies that the addition of Mn–Co nanoparticles did not affect the MOF structure. The detection of electronic interactions between the nanoparticles and MIL-100 is demonstrated by small variations in the absorption intensity and the location of the edges.^36,37^ The optical band gap of Mn–Co@MIL-100 was calculated using the Tauc plot, and it was found to be 1.85 eV. The trend in band gaps was as follows: Mn–Co bimetallic nanoparticles > Mn–Co@MIL-100 > MIL-100. The intermediate band gap of Mn–Co@MIL-100 further demonstrates the electronic interaction between Mn–Co nanoparticles and MIL-100(Fe), confirming the synthesis of hetero-interfaces. These hetero-interfaces can facilitate charge transfer and charge separation within the composite.^5,39,41^

### 3.2 Fourier-transform infra-red spectroscopy

The Fourier-Transform Infra-Red spectra of Mn–Co nanoparticles, MIL-100(Fe), and Mn–Co@MIL-100 were recorded in the 500–4000 cm^−1^ range to investigate the functional group and metal oxygen interactions and presented in the Fig. 3, respectively. The broad absorption band observed in the 2700–3700 cm^−1^ range in all the three samples can be attributed to the O–H vibrations of surface hydroxyl groups and adsorbed H_2_O molecules, indicating the presence of physically adsorbed moisture.^42–45^

**Fig. 3:**
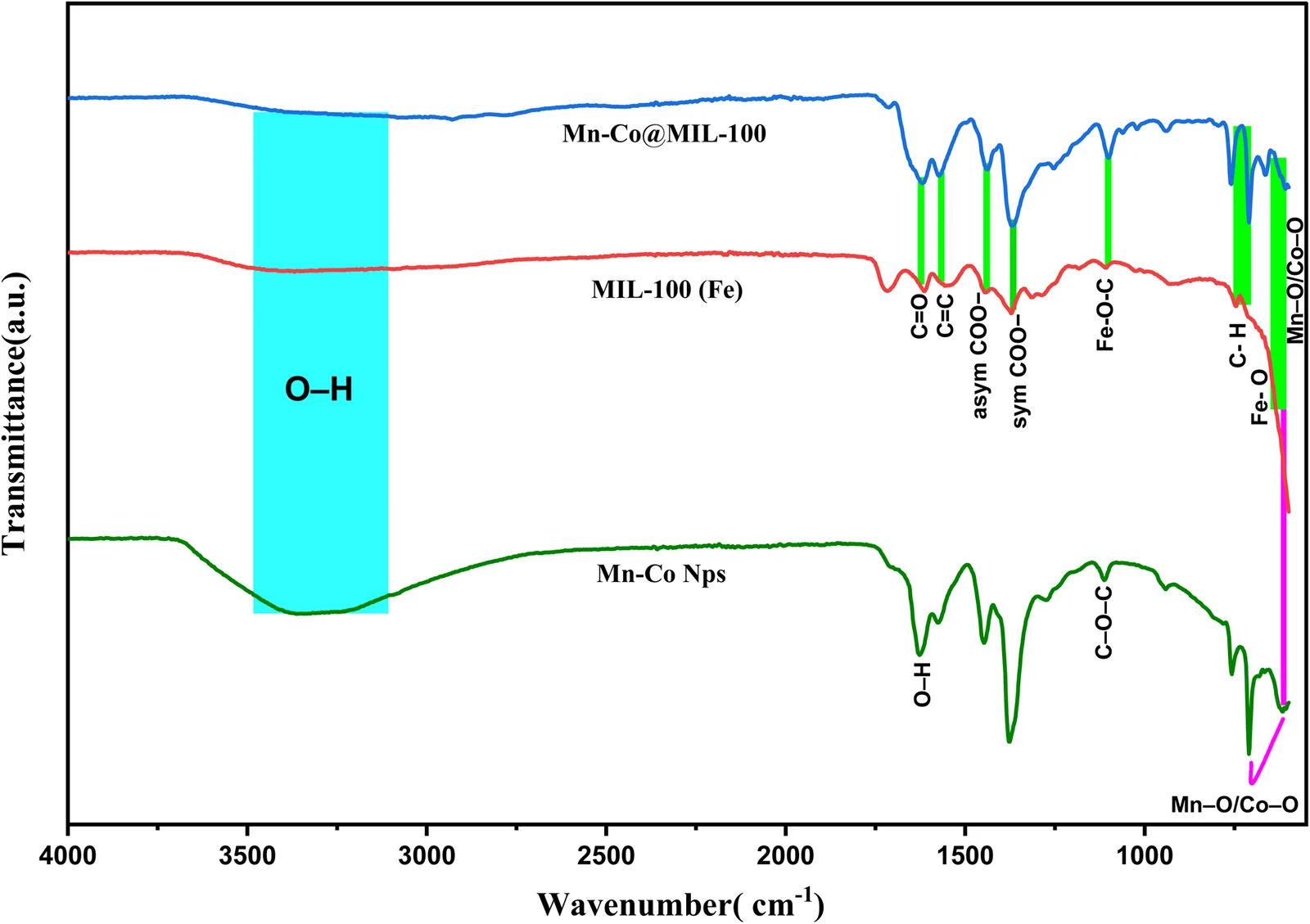
FTIR analysis of Mn–Co NPs, MIL-100 (Fe), and Mn–Co@MIL-100 composite showing characteristic functional groups and metal–oxygen vibrations.

Although the bands at 1349 and 1101 cm^−1^ are associated with C–O–C stretching of PEG, indicating its role as a capping agent, the unique peaks at approximately 1630 cm^−1^ in Mn–Co nanoparticles are associated with O–H bending vibrations.^46,47^ The presence of two high absorption bands in the lower wavenumber region, particularly at ∼650 cm^−1^ and ∼560 cm^−1^, is assigned to Co–O and Mn–O stretching vibrations, confirming the successful formation of bimetallic oxide nanoparticles.^48,49^

The presence of organic linkers in MIL-100(Fe) is confirmed by distinct peaks at 1625 cm^−1^ due to C=O stretching, 1561 cm^−1^ corresponding to C=C vibrations of the benzene ring, 1441 and 1370 cm^−1^ attributed to the asymmetric and symmetric stretching of COO^−^ groups.^50^ Moreover, the peak at the frequencies of approximately 618 cm^−1^ due to Fe–O vibrations and the band at 1109 cm^−1^ due to the Fe–O–C bond are reported to confirm the development of the MIL-100 structure.^51,52^

The FTIR spectrum of Mn–Co MIL-100 after incorporation reveals that the majority of the typical peaks of MIL-100 are still retained relatively, showing that the framework structure has not significantly changed. However, the formation of new bands in the low wavenumber (∼600–660 cm^−1^) related to Mn–O/Co–O and Fe–O–M vibrations proves the successful integration of Mn and Co into the MIL-100 structure. There is no significant shift in peaks or considerable addition of Fe–O and Mn–O/Co–O to the framework, which means that the bimetallic species are introduced to the framework without affecting the structural integrity.^48^

### 3.3 X-ray diffraction analysis

The XRD patterns of MIL-100(Fe) and the Mn–Co@MIL-100 composite are shown in Fig. 4. The MIL-100(Fe) diffraction pattern confirms the effective formation of a well-crystalline framework structure. The formation of the typical MIL-100 porous architecture is verified by the specific peaks observed at about 10.7°, 12.5° and 14.3° (2*θ*), which are the (113), (333) and (791) planes, respectively, as reported in the literature,^53–58^ confirming the formation of the typical MIL-100 porous architecture. Moreover, the poor reflections at around 42.6° and 46° are related to the (100) and (002) planes of the Fe species, respectively, in line with the JCPDS card No. 00-034-0529.

**Fig. 4:**
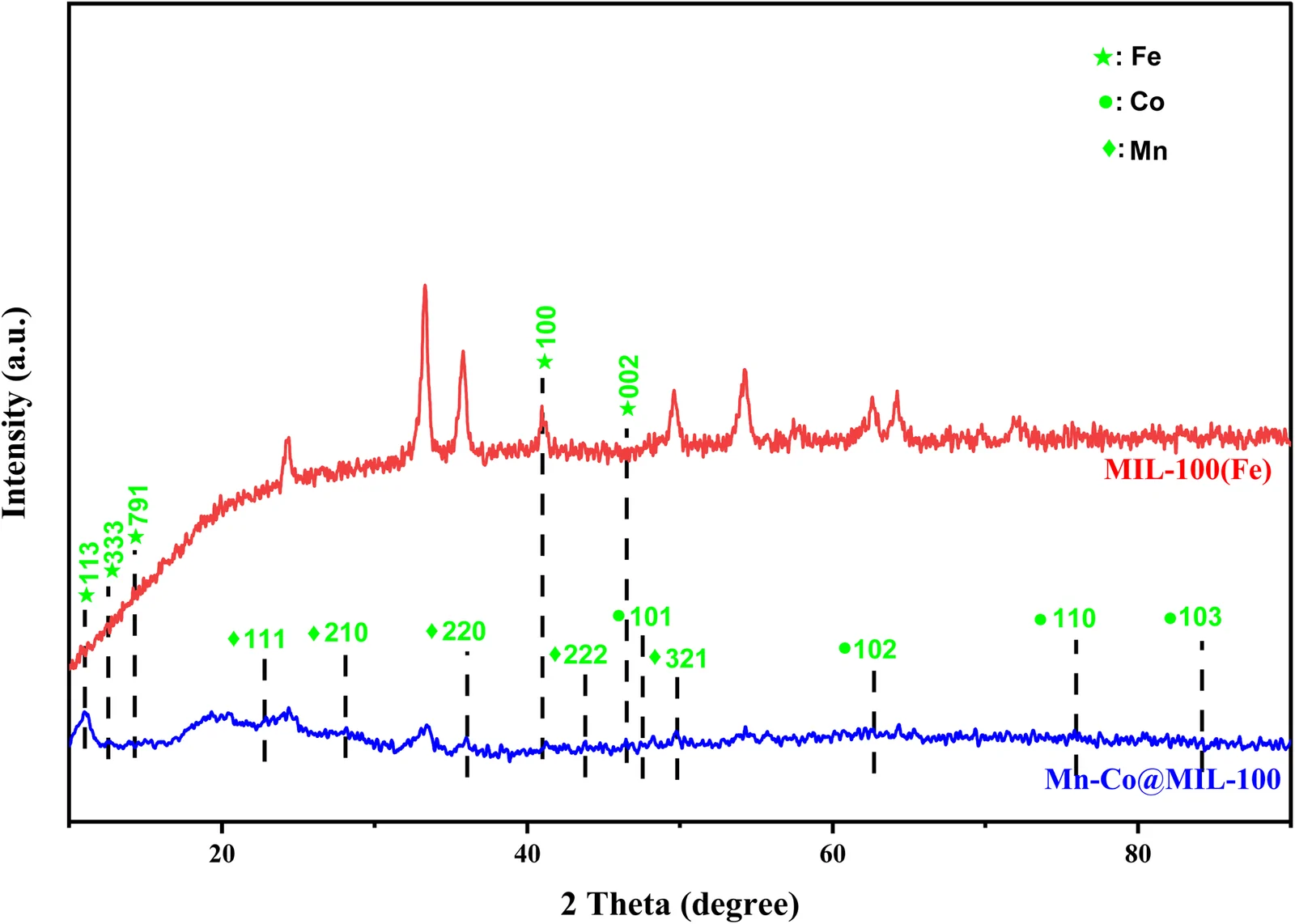
XRD patterns of MIL-100(Fe) and Mn–Co@MIL-100.

The diffraction peaks at 10.95°, 12.5° and 14.3° corresponding to the (113), (333) and (791) planes indicates that the Mn–Co@MIL-100 composite retains the key structural features of MIL-100 after the incorporation of metals. Nonetheless, slight shifts in the broadening and intensity of the peaks give an indication of structural change due to the incorporation of metals. Fe-related planes indexed with JCPDS No. 00-034-0529 are also linked to the peaks at around 42.6° (100) and 46° (002). The peaks appeared at 22.03° (111), 28.18° (210), 35.99° (220), 44.78° (222), 47.56° (101), 49.82° (321), 62.72° (102), 75.94° (110), 84.19° (103), and 90.62° (200) are a confirmation of the successful integration of metal species into the MIL-100 structure and related to oxide phases of Mn and Co and are well aligned with JCPDS No. 00-021-0547 and 00-005-0727, respectively.

### 3.4 Structural and morphological studies

We used SEM and EDX to compare the surface morphology and elemental composition of pure MIL-100(Fe) and the Mn–Co@MIL-100 composite (Fig. 5). The SEM micrographs of MIL-100 exhibit predominantly a spherical morphology with slight agglomeration, in which closely spaced particles form and connect to give the surface a rough and porous texture. Due to its higher surface area and greater ion transportability, such morphology, which is characteristic of MOFs, is beneficial in electrochemical applications.^59–61^ Conversely, after being included, the Mn–Co@MIL-100 composite exhibits morphological changes that are identifiable. The structure transforms into crystalline traits with partly fractured particles that are irregularly polyhedral and plate-like. Also, there are many small nanoparticles that are to some extent agglomerated in certain regions that decorate the surface of the metal organic framework crystals. This surface modification is an indication of the successful deposition of Mn–Co bimetallic species and the formation of hetero-interfaces between the nanoparticles and the MIL-100 framework. These structural features are expected to increase the number of active sites and improve the electrochemical performance.

**Fig. 5:**
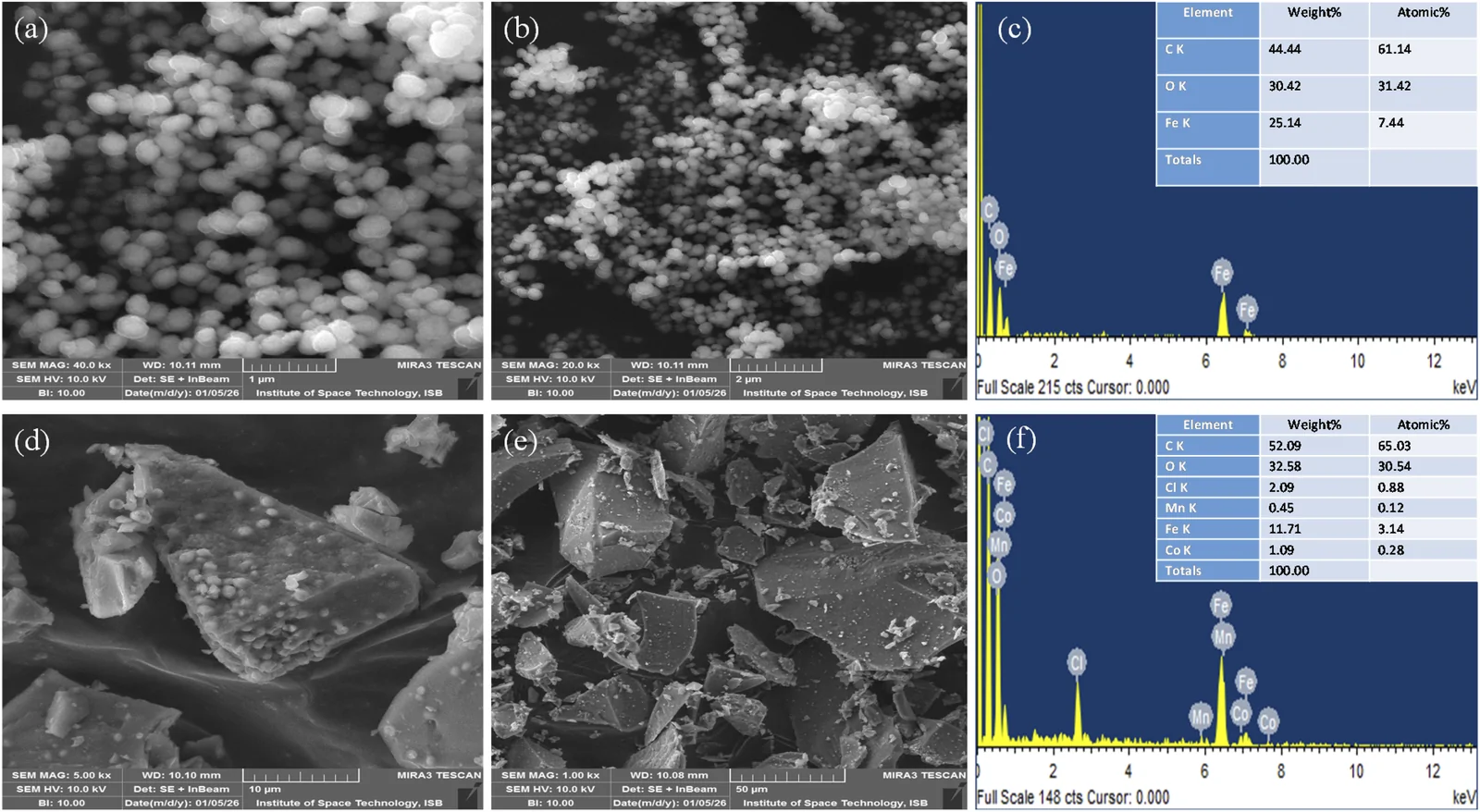
SEM micrographs of MIL-100 (a and b) and Mn–Co@MIL-100 (c and d) recorded at different magnifications, along with EDX spectra of (e) MIL-100 and (f) Mn–Co@MIL-100 confirming the elemental composition of the synthesized materials.

The elemental composition of both compounds was further confirmed by analysis of EDX. The MIL-100 spectrum proves the presence of carbon, oxygen, and iron. Formation of the Fe-based structure is supported by the emergence of unique peaks at regions of approximately 0.26 keV (C), 0.52 keV (O), and 6.4–7.0 KeV (Fe). Based on the predicted composition of MIL-100 that is predicted, the percentages of weight of C, O, and Fe are approximately 44.44 wt%, 30.42 wt%, and 25.14 wt%.^40^ After incorporation, the EDX spectrum of Mn–Co@MIL-100 exhibits additional peaks of cobalt (Co) and manganese (Mn), and C, O, Fe, and some chlorine (Cl) signal. The presence of the Mn, Fe, and Co peaks at around 5.9 keV, 6.4 keV, and 6.9 keV, respectively, is an indication of the successful integration of the bimetallic species into the framework. The elemental composition is made up of approximately 52.09 wt% C, 32.58 wt% O, 11.71 wt% Fe, 0.45 wt% Mn, 1.09 wt% Co, and 2.09 wt% Cl. The primary MIL-100 elements remain, and the presence of Mn and Co confirms that incorporation was done without causing any significant contaminants.

### 3.5 Brunauer–Emmett–Teller (BET) analysis

The textural properties of the synthesized MIL-100 were studied using nitrogen adsorption–desorption analysis using the BET method, and results are shown in Table 1. The BET study indicates that a specific surface area of 17.714 m^2^ g^−1^ was obtained at the linear area of the adsorption isotherm, having a correlation coefficient (*r* = 0.934). The relatively medium-sized BET surface area might be attributed to the partial blockage of the pores or the modifications of the structure that occur during the synthesis process and consequently restrict the access of nitrogen molecules to the internal pores of the framework. Similar decreases in the BET surface area have been reported in modified or doped MOF systems in which guest species fill pore cavities and lower the number of available adsorption sites for nitrogen molecules. Such structural variations are capable of influencing the identified surface area without completely eliminating the intrinsic porosity of the framework.^62–64^

**Table 1:** BET analysis of Mn–Co@MIL-100 composite

Parameter	Value	Unit	Method
BET surface area	17.717	m^2^ g^−1^	Multi-point BET (*r* = 0.934)
Langmuir surface area	23.693	m^2^ g^−1^	Langmuir model (*r* = 0.949)
Micropore surface area	124.295	m^2^ g^−1^	*t*-Plot (de Boer)
Micropore volume	0.063	cm^3^ g^−1^	*t*-Plot (de Boer)
Total pore volume (pores <2.6 nm)	8.815 × 10^−3^	cm^3^ g^−1^	Single point adsorption at *P*/*P*_0_ = 0.30475

Further analysis using the Langmuir adsorption model gave a surface area of 23.693 m^2^ g^−1^ with a correlation coefficient of 0.949 that is slightly higher than the BET surface area. As the Langmuir model supposes that the monolayer adsorption is perfect on a uniform surface, this finding is a common one in adsorption studies. The Langmuir model therefore often over estimates the surface area as compared to the BET model. Nevertheless, the concordance of the two models means that the material structure has available adsorption sites,^65,66^ as summarized in Table 1.

Further analysis of the pore properties was done with the *t*-plot (DeBoer) method. The *t*-plot analysis established a micropore volume of 0.063 cm^3^ g^−1^ and a micropore surface area of 124.295 m^2^ g^−1^, which means that the framework has significant microporous areas. In the case of pores that were smaller than 2.6 nm, the total pore volume was determined as 8.815 × 10^−3^ cm^3^ g^−1^at a relative pressure (*P*/*P*_0_ = 0.30475). These results confirm the existence of microporous channels in the material that can facilitate accessibility of electrolytes and diffusion of ions. The presence of the micropores in the electrochemical energy storage materials is also vital even in the case of the presence of quite moderate observed surface area to enhance the interaction between the electrodes and electrolyte and the possibility of charging storage.^67^ Also, in the situations where the observed surface area is moderate, the transition metal-based electrode materials may also serve to store pseudocapacitive charges by further enhancing capacitance due to redox reactions.^68–70^

It should be noted that the micropore volume obtained from the *t*-plot method (0.063 cm^3^ g^−1^) and the total pore volume for pores smaller than 2.6 nm obtained from single-point adsorption analysis (8.815 × 10^−3^ cm^3^ g^−1^ at *P*/*P*_0_ = 0.30475) are derived using different calculation approaches applied at different regions of the adsorption isotherm, and are therefore not directly comparable as nested quantities.

### 3.6 Electrochemical performance

The electrochemical behavior of both pristine MIL-100(Fe) and the Mn–Co/MIL-100(Fe) nanocomposite electrodes was investigated by cyclic voltammetry (CV) in a three-electrode configuration using 1 M KOH aqueous electrolyte. CV measurements were carried out over a potential window of 0–0.8 V at scan rates ranging from 5 to 50 mV s^−1^, and the resulting voltammograms are presented in Fig. 6a and b for MIL-100(Fe) and Mn–Co/MIL-100(Fe), respectively. For both electrodes, the current response and the enclosed area of the CV curves increased progressively with increasing scan rate, confirming good electrochemical activity and capacitive charge storage in both materials.^71^ The pristine MIL-100(Fe) electrode (Fig. 6a) exhibited relatively broad, weak anodic humps in the 0.55–0.7 V region with poorly resolved cathodic counterparts, and a comparatively low current density (maximum ∼0.017 A cm^−2^ at 50 mV s^−1^). This diffuse, quasi-rectangular voltammetric response indicates a predominantly weak pseudocapacitive contribution from the Fe centers in the unmodified framework, consistent with the intrinsically low electrical conductivity and limited density of accessible redox-active sites in single-metal MIL-100(Fe).^72,73^ In marked contrast, the Mn–Co/MIL-100(Fe) nanocomposite (Fig. 6b) displayed considerably more prominent and well-defined redox peaks, with a distinct anodic peak near ∼0.65–0.75 V and a corresponding cathodic peak near ∼0.3–0.35 V, together with a substantially larger current response (maximum ∼0.04 A cm^−2^) and enclosed CV area relative to pristine MIL-100(Fe) under identical conditions. This pronounced enhancement confirms that incorporation of Mn–Co bimetallic sites introduces additional, more clearly resolved faradaic redox activity attributable to the reversible Mn^2+^/Mn^3+^ and Co^2+^/Co^3+^ transitions that is largely absent or strongly suppressed in the pristine framework. The same class of electrochemical behavior (broad, weak redox features in single-metal MOFs *versus* sharper, higher-current redox couples in bimetallically doped analogues) has been reported for various MOF- and transition-metal-oxide-based supercapacitor electrodes.

**Fig. 6:**
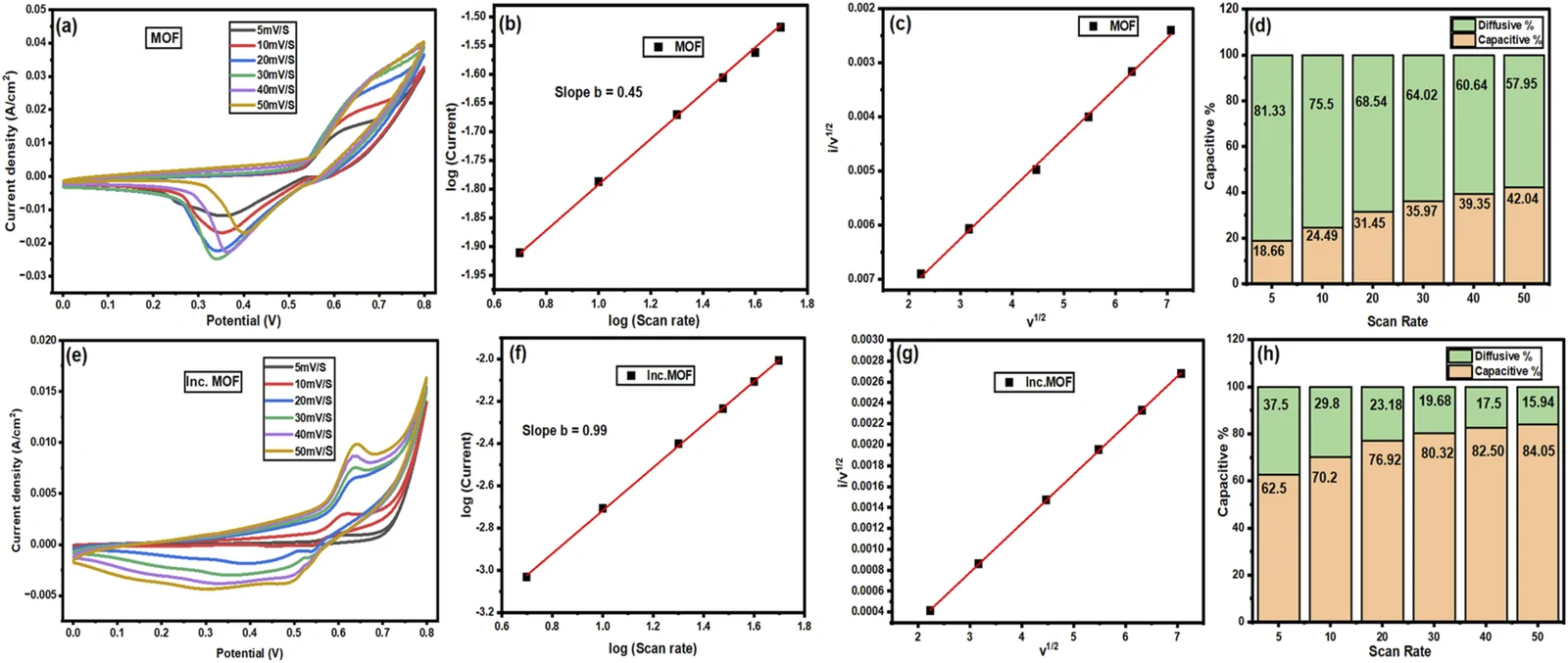
Charge-storage kinetics analysis of pristine MIL-100(Fe) (a–d) and Mn–Co/MIL-100(Fe) nanocomposite (e–h). (a and e) CV curves at scan rates of 5–50 mV s^−1^; (b and f) corresponding log(*i*) *vs.* log(*ν*) plots used to determine the *b*-values (0.45 for MIL-100(Fe) and 0.99 for Mn–Co/MIL-100(Fe)); (c and g) *i*/*ν*^1/2^*vs. ν*^1/2^ plots used in Dunn's method to separate capacitive and diffusive contributions; (d and h) bar charts showing the relative capacitive (orange) and diffusive (green) contributions to the total current at each scan rate for MIL-100(Fe) and Mn–Co/MIL-100(Fe), respectively.

Even at higher scan rates, both electrodes largely retained their characteristic CV shape, indicating reasonable structural stability and electrochemical reversibility during cycling; however, the composite's redox peaks remained visible and better resolved across the full scan-rate range, whereas the pristine MIL-100(Fe) humps became increasingly diffuse at higher scan rates, suggesting more sluggish charge-transfer kinetics in the unmodified framework. Overall, this direct comparison under identical experimental conditions demonstrates that Mn–Co bimetallic incorporation substantially enhances the faradaic redox activity and current response of the MIL-100(Fe) framework, providing clear electrochemical evidence for the synergistic contribution of the Mn–Co active sites to the improved capacitive performance of the nanocomposite.

To further clarify the charge-storage mechanism of pristine MIL-100(Fe) and the Mn–Co/MIL-100(Fe) nanocomposite, the relationship between peak current (*i*) and scan rate (*ν*) was analyzed using the power-law expression:

3 *i* = *av*^*b*^

where *a* and *b* are adjustable parameters, and *b* is obtained from the slope of the linear plot of log(*i*) *versus* log(*ν*) (Fig. 6b and f). A *b*-value close to 0.5 indicates a diffusion-controlled charge-storage process, whereas a value approaching 1.0 indicates a predominantly surface-controlled (capacitive) mechanism.

For pristine MIL-100(Fe), the calculated *b*-value was 0.45 (Fig. 6b), close to the diffusion-controlled benchmark of 0.5, indicating that charge storage in the unmodified framework is governed predominantly by ion diffusion into the porous structure rather than by fast surface faradaic reactions consistent with the relatively limited electrical conductivity and restricted accessibility of electroactive Fe sites in the parent MOF. In marked contrast, the Mn–Co/MIL-100(Fe) nanocomposite exhibited a substantially higher *b*-value of 0.99 (Fig. 6f), approaching the ideal surface-controlled limit of 1.0, indicating that charge storage in the bimetallic composite is overwhelmingly governed by fast, surface-confined capacitive/pseudocapacitive processes rather than by sluggish solid-state ion diffusion. This marked shift from diffusion-controlled (MIL-100(Fe)) to predominantly surface-controlled (Mn–Co/MIL-100(Fe)) behavior directly demonstrates that incorporation of Mn–Co bimetallic active sites improves charge-transfer kinetics and enhances the accessibility of electroactive surface sites within the MOF framework, providing clear kinetic evidence for the beneficial role of bimetallic doping.

To further quantify and validate this mechanistic distinction, the relative capacitive and diffusive contributions to the total current response were separated using Dunn's method:

4 *i*(*V*) = *k*_1_*v* + *k*_2_*v*^1/2^

where *k*_1_*v* represents the surface-capacitive contribution and *k*_2_*v*^1/2^ represents the diffusion-controlled contribution, determined from the slope and intercept of the linear plot of *i*/*ν*^1/2^*versus ν*^1/2^ (Fig. 6c and g). For pristine MIL-100(Fe) (Fig. 6d), the capacitive contribution increased modestly from 18.66% at 5 mV s^−1^ to 42.04% at 50 mV s^−1^, with the diffusion-controlled process remaining dominant (57.95–81.33%) across the entire scan-rate range, confirming the diffusion-limited nature of charge storage indicated by its *b*-value. For the Mn–Co/MIL-100(Fe) nanocomposite (Fig. 6h), the capacitive contribution was substantially higher at every scan rate, increasing from 62.5% at 5 mV s^−1^ to 84.05% at 50 mV s^−1^, confirming that charge storage in the bimetallic composite is predominantly surface-controlled, in excellent agreement with its high *b*-value (0.99). As expected, the capacitive contribution increased with scan rate for both electrodes, since diffusion-limited processes have progressively less time to occur at higher sweep rates, allowing the faster surface-capacitive process to dominate the overall current response.

Taken together, the *b*-value and Dunn's-method analyses provide consistent, complementary evidence that Mn–Co bimetallic incorporation fundamentally alters the charge-storage mechanism of the MIL-100(Fe) framework shifting it from a predominantly diffusion-controlled process in the pristine material to a predominantly surface-controlled, pseudocapacitive process in the nanocomposite and directly substantiates the improved charge-transfer kinetics responsible for the enhanced electrochemical performance of Mn–Co/MIL-100(Fe) relative to pristine MIL-100(Fe).

Galvanostatic charge–discharge (GCD) measurements were performed for both pristine MIL-100(Fe) and the Mn–Co/MIL-100(Fe) nanocomposite at current densities ranging from 0.8 to 2 A g^−1^, as shown in Fig. 7a and c, respectively. Both electrodes displayed non-linear, asymmetric potential-time profiles rather than ideal triangular shapes, consistent with a faradaic, pseudocapacitive charge-storage mechanism arising from reversible redox transitions at the electroactive metal centers, in agreement with the CV results. MIL-100(Fe) was cycled within a 0–0.6 V window, while Mn–Co/MIL-100(Fe) was cycled within 0–0.5 V, and both electrodes showed progressively shorter discharge times with increasing current density, consistent with reduced ion-diffusion time and correspondingly lower accessible capacity at higher charge–discharge rates.

**Fig. 7:**
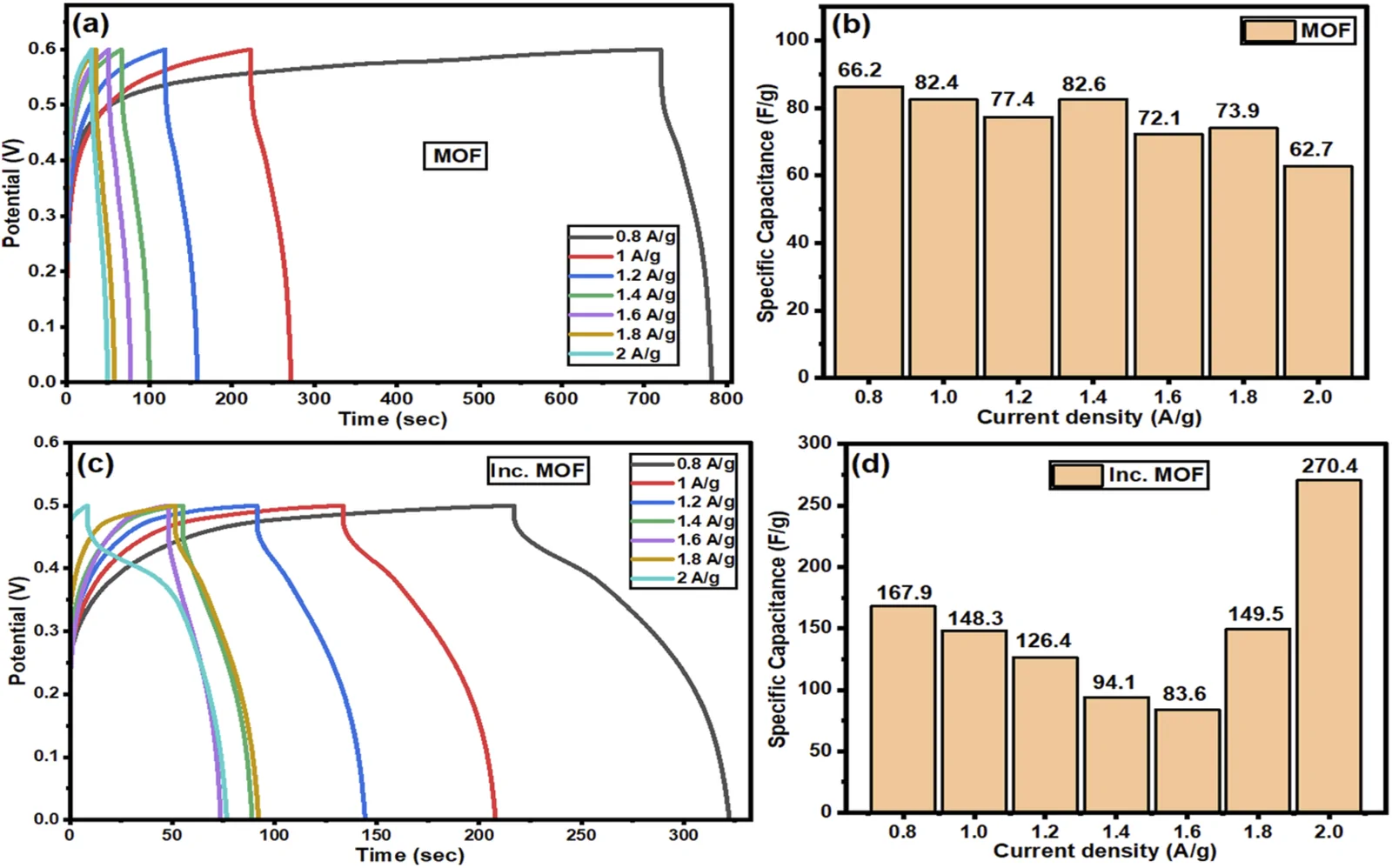
Galvanostatic charge–discharge (GCD) analysis of pristine MIL-100(Fe) (a and b) and Mn–Co/MIL-100(Fe) nanocomposite (c and d). (a and c) GCD profiles at current densities of 0.8–2.0 A g^−1^; (b and d) corresponding specific capacitance (*C*_s_) as a function of current density.

To calculate the specific capacitance (*C*_s_, F g^−1^) of the discharge curves, eqn (5) was used:^74^5
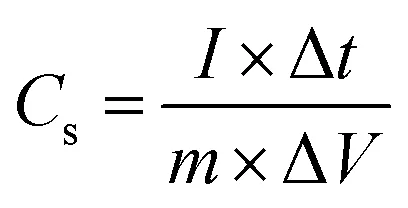
Here *C*_s_ is the specific capacitance (F g^−1^), *I* is the discharge current (A), Δ*t* is the discharge time (s), *m* is the mass of active material (g) and Δ*V* is the working potential window (V).

The specific capacitance (*C*_s_) was calculated from the GCD discharge curves using eqn (5). As shown in Fig. 7b, pristine MIL-100(Fe) delivered a specific capacitance of 66.2 F g^−1^ at 0.8 A g^−1^, fluctuating moderately between 62.7 and 82.6 F g^−1^ across the 0.8–2.0 A g^−1^ range. In contrast, the Mn–Co/MIL-100(Fe) nanocomposite (Fig. 7d) delivered a substantially higher specific capacitance of 167.9 F g^−1^ at 0.8 A g^−1^ approximately 2.5-fold greater than pristine MIL-100(Fe) at the same current density directly confirming that Mn–Co bimetallic incorporation significantly enhances the charge-storage capacity of the MIL-100(Fe) framework under identical measurement conditions. The composite's capacitance decreased with increasing current density from 0.8 to 1.6 A g^−1^ (167.9 to 83.6 F g^−1^), consistent with the typical rate-dependent behavior of pseudocapacitive electrodes, in which only a fraction of the electroactive sites can participate in redox reactions at faster charge–discharge rates due to limited ion-diffusion time^75^ This enhancement in specific capacitance for the Mn–Co/MIL-100(Fe) nanocomposite relative to pristine MIL-100(Fe), obtained under identical electrolyte, mass-loading, and measurement conditions, provides direct, quantitative evidence that incorporation of Mn and Co redox-active centers into the MIL-100(Fe) framework enhances the density of accessible faradaic charge-storage sites, consistent with the marked shift toward surface-controlled, capacitive-dominated behavior revealed by the *b*-value and Dunn's-method analyses (Fig. 6).^76–78^

Electrochemical impedance spectroscopy (EIS) was used to investigate the charge transfer properties and ion transport behavior of the synthetic electrode material. The Nyquist plot is shown in Fig. 8. The coexistence of charge transfer resistance and ion diffusion processes was shown by the low-frequency region's inclined line and the high-frequency region's little depressed semicircle.

**Fig. 8:**
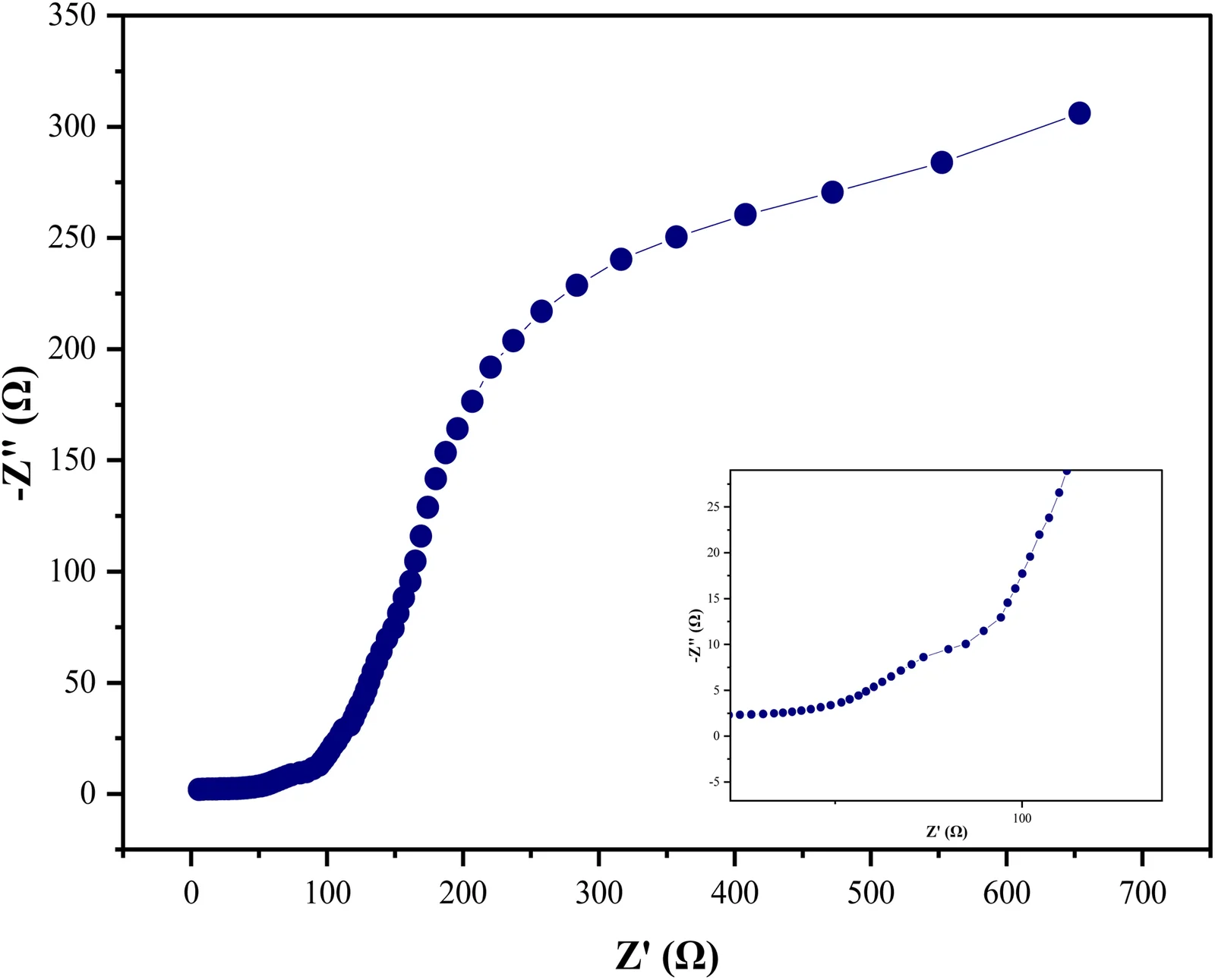
Nyquist plot of Mn–Co@MIL-100 electrode.

The *R*_s_ was recorded from the Nyquist plot at high frequency on the real axis (*Z*′), and the value of *R*_s_ was found to be 53.10 Ω. The resulting *R*_s_ value provides information on the electrical conductivity and interfacial interaction with the active material, the current collector and the electrolyte. It is also an intrinsic resistance of the electrode/electrolyte system. In the high frequency zone, diameter of the semicircle was used to calculate the *R*_ct_ and it was found that it is 31.87 Ω. The value of *R*_ct_ is confirmed to be related to the faradaic charge storage activities at the electrode/electrolyte interface.^79,80^

Also, the slanted line in the low frequency range corresponds to the Warburg impedance caused by the movement of electrolyte ions inside the porous structure of the electrode. A diffusion-controlled zone suggests the presence of ion diffusion processes and of transport of the electrolytes in the porous framework of the electrode. The electrochemical impedance spectroscopy data indicates that the overall charge transfer behaviour and diffusion properties of the site are good for the Mn–Co@MIL-100 electrode. Based on the reported resistances, its electrochemical performance can be further enhanced by increasing its electrical conductivity.

### 3.7 Cycling stability of Mn–Co/MIL-100(Fe) composite

The cycling stability of the Mn–Co/MIL-100(Fe) nanocomposite electrode was evaluated by repeated galvanostatic charge–discharge cycling over 52 cycles, with the specific capacitance and coulombic efficiency plotted as a function of cycle number in Fig. 9. Rather than displaying the capacitance decay typically associated with electrode degradation, the Mn–Co/MIL-100(Fe) electrode exhibited a progressive increase in specific capacitance over the cycling period, rising from ∼98 F g^−1^ in the initial cycles to ∼170 F g^−1^ by the 52nd cycle. This behavior is consistent with an electrochemical “activation” effect commonly observed in MOF- and transition-metal-based pseudocapacitive electrodes, wherein repeated cycling progressively improves electrolyte penetration into the porous framework and enhances the accessibility of internal redox-active Mn/Co sites that are not fully wetted or activated during the initial cycles.

**Fig. 9:**
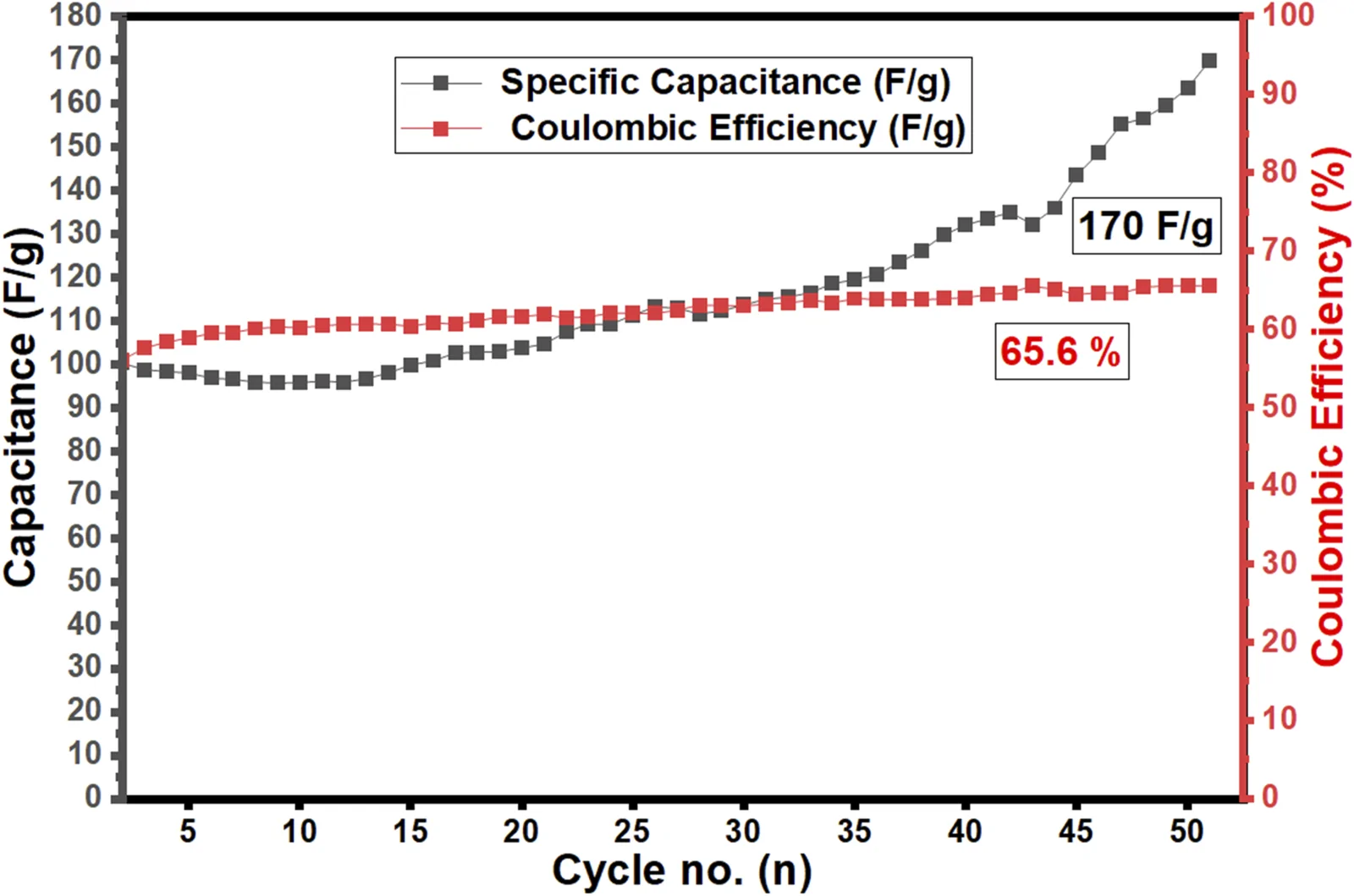
Cycling stability of the Mn–Co/MIL-100(Fe) nanocomposite electrode over 52 charge–discharge cycles, showing specific capacitance (black, left axis) and coulombic efficiency (red, right axis) as a function of cycle number.

The coulombic efficiency remained relatively stable throughout cycling, increasing modestly from ∼56% in the early cycles to ∼65.6% by the 52nd cycle, indicating progressively improving reversibility of the faradaic charge-storage reactions as the electrode structure is electrochemically activated. Although these results were obtained over a comparatively short cycling window (52 cycles) due to time constraints, they show no evidence of capacitance fading or structural degradation over this period; rather, the consistent upward trend in both capacitance and coulombic efficiency suggests good short-term electrochemical stability and progressive activation of the Mn–Co/MIL-100(Fe) electrode. Extended long-term cycling studies (hundreds to thousands of cycles) will be necessary to fully establish the long-term capacitance retention and structural durability of this electrode material, and are identified as a priority for future work.

### 3.8 MG dye removal study

Adsorption experiments were conducted at the natural pH of the MG solution rather than at externally adjusted pH values. This choice is consistent with previous reports on MG dye removal using various adsorbents including metal oxide nanoparticles and natural biomass-based materials which have generally identified pH ∼6–7 as the optimal range for maximum MG removal, owing to favorable electrostatic interactions between the cationic dye and the deprotonated/negatively charged adsorbent surface in this pH window.^81–83^ The adsorptive removal % of malachite green (MG) dye was evaluated by monitoring the characteristic absorbance peak at *λ*_max_ = 615 nm using UV-vis spectroscopy (Fig. 10). The blank MG solution exhibited a strong, well-defined peak at 615 nm (*A*_0_ = 0.11209), confirming the dye's stability in the absence of adsorbent. After 3 h of contact time, pristine MIL-100(Fe) showed only a modest decrease in absorbance (*A*_*t*_ = 0.09347), corresponding to a removal efficiency of ∼16.61%, indicating limited intrinsic adsorption removal consistent with the relatively weak surface interaction and moderate porosity of the unmodified framework.

**Fig. 10:**
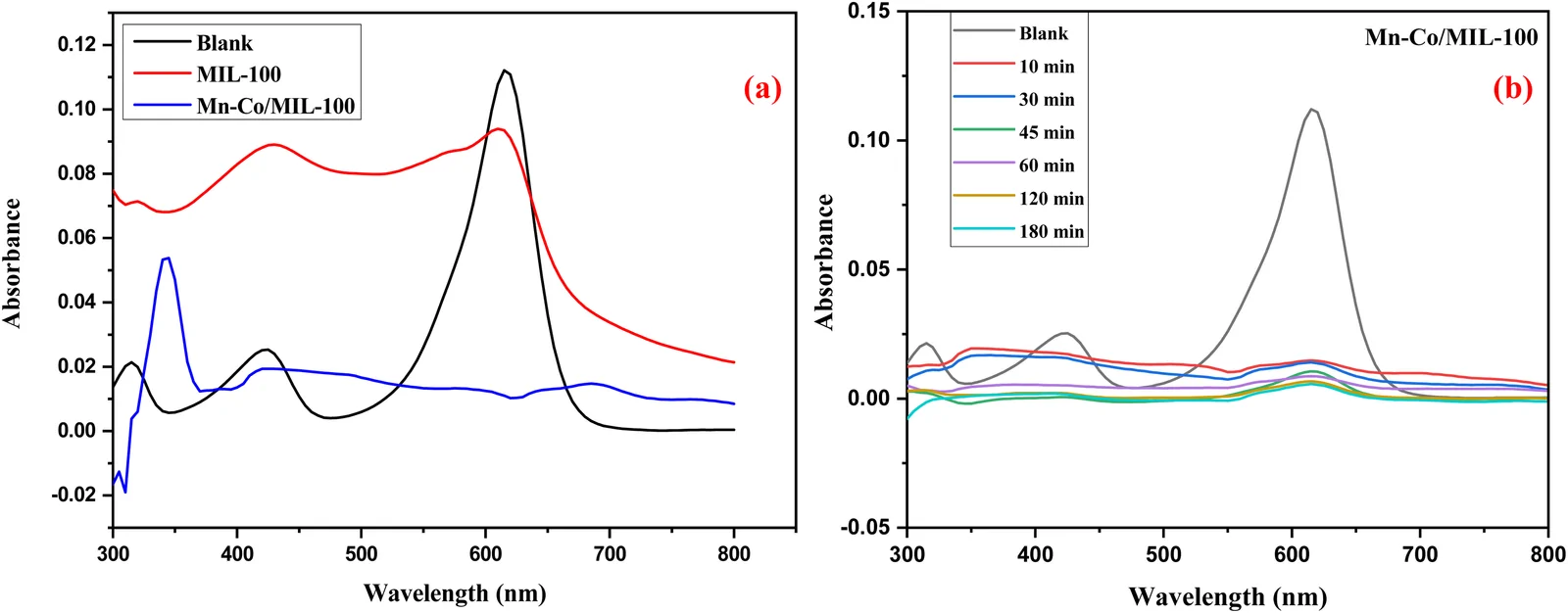
(a) UV-vis absorption spectra showing MG dye removal by pristine MIL-100(Fe) and Mn–Co/MIL-100(Fe) after 3 h contact time; Mn–Co/MIL-100(Fe) exhibits markedly superior percentage removal (∼90.18%) compared to pristine MIL-100(Fe) (∼16.61%), while (b) time-dependent UV-vis spectra showing MG removal by Mn–Co/MIL-100(Fe) at increasing contact times (10–180 min) *versus* blank MG.

In contrast, the Mn–Co/MIL-100(Fe) nanocomposite exhibited a dramatic suppression of the 615 nm peak (*A*_*t*_ = 0.01101), achieving a removal efficiency of ∼90.18% under identical conditions. This near-complete disappearance of the characteristic MG chromophore band confirms the substantially enhanced adsorptive removal efficiency imparted by Mn–Co bimetallic incorporation in the MOF structure.

This enhancement in removal efficiency relative to pristine MIL-100(Fe) can be attributed to several synergistic factors such as the introduction of additional Lewis acidic Mn^2+^/Co^2+^ sites that promote stronger electrostatic and coordinative interactions with the cationic MG dye molecules; and increased surface defect density and possible mesoporosity arising from bimetallic nanoparticle incorporation, improving accessibility of active adsorption sites. Notably, the full-scan spectra (300–800 nm) also reveal that Mn–Co/MIL-100(Fe) suppresses not only the primary 615 nm band but also the secondary bands at ∼420 nm and ∼480 nm associated with MG's conjugated structure, with all characteristic bands diminishing proportionally and without the appearance of new peaks or spectral shifts, confirming that the intact MG dye molecule is adsorbed onto the nanocomposite surface rather than undergoing chemical degradation of its chromophoric system.

### 3.9 Kinetic study

To understand the rate-limiting mechanism of MG dye removal by the Mn–Co/MIL-100(Fe) nanocomposite, the time-dependent adsorption data (10–180 min), Fig. 11a and b were fitted to the pseudo-first-order (8) and pseudo-second-order (9) kinetic models:8
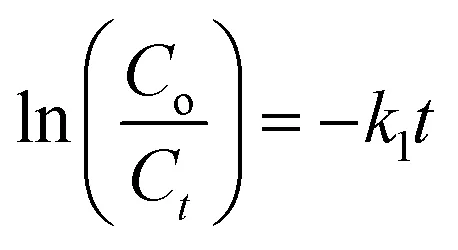
9
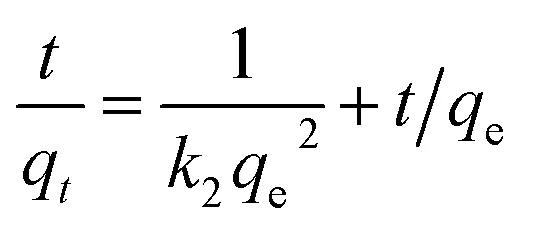


The linear fits are shown in Fig. 11a and b, and the derived parameters are summarized in Table 2. The first order model yielded a moderate correlation (*k*_1_ = 0.00584 min^−1^, *R*^2^ = 0.8849), whereas the second order model provided an excellent linear fit (*k*_2_ = 0.02335 g mg^−1^ min^−1^, *R*^2^ = 0.9998). Critically, the 2nd order-calculated equilibrium adsorption capacity (*q*_e_, calc. = 19.20 mg g^−1^) closely matched the experimentally observed value (*q*_e_, exp. = 19.00 mg g^−1^), while the 1st order model failed to give a physically meaningful *q*_e_. These results confirm that MG dye adsorption onto the Mn–Co/MIL-100(Fe) nanocomposite follows pseudo-second-order kinetics, indicating that chemisorption is the rate-limiting step, involving electron sharing/exchange interactions between MG molecules and active surface sites including unsaturated Fe^3+^/Mn/Co metal centers and surface oxygen-containing functional groups.

**Fig. 11:**
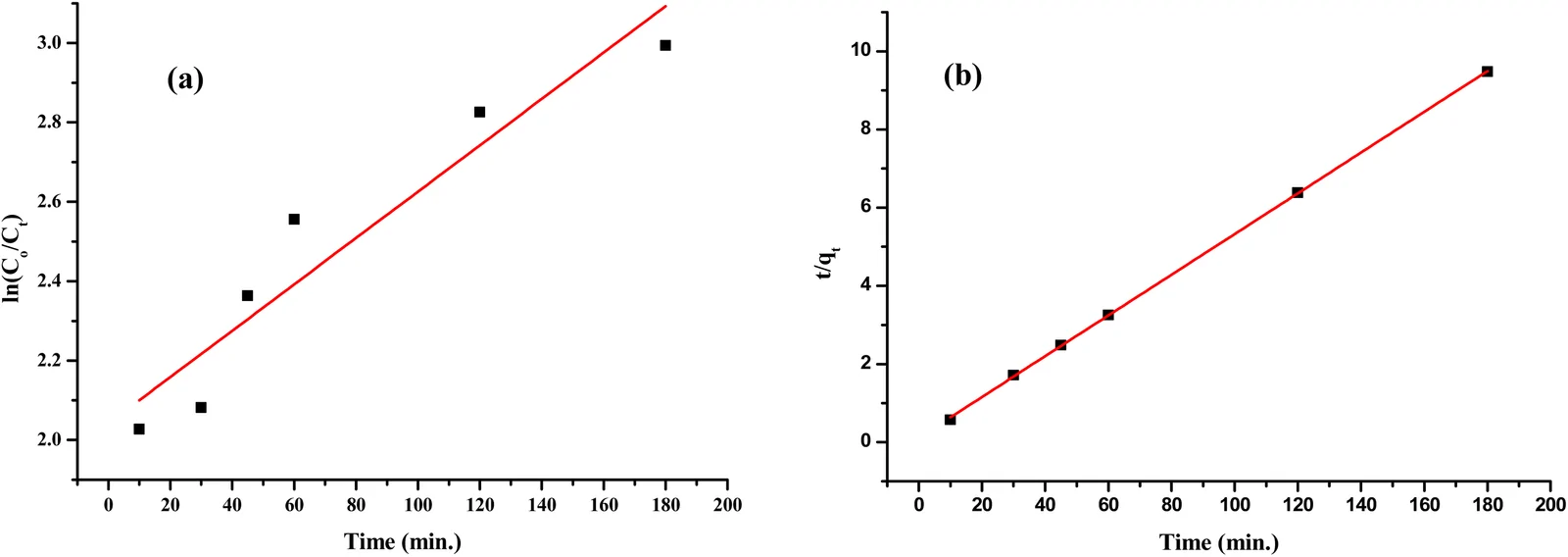
Linear fits of (a) pseudo-first-order and (b) pseudo-second-order kinetic models for the removal of MG dye by Mn–Co/MIL-100(Fe) nanocomposite.

**Table 2:** Kinetic parameters for MG dye adsorption onto Mn–Co/MIL-100(Fe) nanocomposite

Model	Parameter	Value
Pseudo-first-order	*K*_1_ (min^−1^)	0.00584
	Intercept (ln *q*_e_)	2.0416
	*R* ^2^ (adj.)	0.8849
Pseudo-second-order	*q*_e_, calc. (mg g^−1^)	19.20
	*q*_e_, exp. (mg g^−1^)	19.00
	*K*_2_ (g mg^−1^ min^−1^)	0.02335
	*R* ^2^ (adj.)	0.9998

This finding is consistent with the modest BET (multi-point) surface area of only 17.717 m^2^ g^−1^ obtained for the Mn–Co/MIL-100(Fe) nanocomposite (Table 1). Notably, the micropore surface area (*t*-plot, 124.295 m^2^ g^−1^) and micropore volume (0.063 cm^3^ g^−1^) are substantially higher than the BET surface area, suggesting that decoration with Mn–Co species partially blocks the mesoporous framework surface while a significant fraction of microporosity is retained internally. Given this constrained external surface area, MG dye removal is unlikely to be governed primarily by pore-diffusion-controlled physisorption; instead, the dominance of 2nd order kinetics indicates that MG uptake proceeds mainly through chemical interaction at accessible, chemically reactive Fe/Mn/Co sites rather than through simple physical adsorption across a large surface area. This settles the comparatively low BET surface area of the composite with its high dye removal efficiency, and supports a mechanism in which active-site density and reactivity rather than surface area alone control the adsorption rate.

We compared the electrochemical and adsorption performance of the Mn–Co/MIL-100(Fe) nanocomposite with previously reported MIL-100(Fe)-based materials, as summarized in Table 3 (energy-storage applications) and Table 4 (dye/pollutant removal). Table 3 shows that MIL-100(Fe)-based electrodes modified *via* carbonization, conductive-polymer hybridization, or secondary metal/MOF incorporation generally exhibit higher capacitance and improved cycling stability than unmodified MIL-100(Fe); while the present nanocomposite shows comparatively modest capacitance relative to these more extensively engineered composites, it clearly benefits from bimetallic incorporation and points toward conductive-additive hybridization as a promising avenue for further optimization. Table 4 similarly shows that MIL-100(Fe)-based materials achieve high removal efficiencies for various dyes, often *via* catalytic/photocatalytic or oxidant-assisted degradation. In contrast, the present nanocomposite achieves efficient MG removal through adsorption, performing competitively with adsorption-based MIL-100(Fe) systems in the literature. Together, these comparisons underscore the promise of MIL-100(Fe)-based materials with high porosity, tunable bimetallic composition, and enhanced redox activity as multifunctional platforms, with this study uniquely demonstrating dual energy-storage and dye-adsorption functionality within a single Mn–Co/MIL-100(Fe) nanocomposite.

**Table 3:** Comparison of MIL-100-based materials for various energy storage applications, including supercapacitors

Material	Capacitance	Current density	Columbic efficiency	Stability	Synthesis	Application	References
MIL-100, Mn–Co/MIL100(Fe)	62.7 F g^−1^, 270.4 F g^−1^	2000 mA g^−1^	65.6%	52 cycles	Hydrothermal	Supercapacitor	Present study
Derivatized carbon from MIL-100(Fe)	383.1 F g^−1^	100–3000 mA g^−1^	Not reported	∼5000 cycles	Hydrothermal	Supercapacitor	84
FeVO_*x*_ (MIL-100(Fe)-derived composite)	325 mAh g^−1^	50–1000 mA g^−1^	≈95% CE (inferred)	4000 cycles	Green and fast synthesis method	Zinc-ion battery	85
PEO/LiTFSI/a-MIL-100 (Fe)/PVDF	161.8 mAh g^−1^	17–340 mA g^−1^	Not specified	200 cycles	Solution casting method	Lithium metal batteries	86
MIL-100(Fe)	∼70–77 mAh g^−1^	100 mA g^−1^	Stable (not quantified)	100 cycles	Solvothermal	Li ion half-cell batteries	87
MIL-100(Fe) (crystalline)	854 mAh g^−1^	85 mA g^−1^	99.57%	100 cycles	Hydrothermal	Lithium-ion battery	88
PANI/Gr/MIL-100(Fe)	638 F g^−1^	1000 mA g^−1^	Not reported	5000 cycles	Hydrothermal	Supercapacitor	15
TiB_2_@MIL-100(Fe)	330 mAh g^−1^	N/A	Not reported	3 cycles	Hydrothermal	Lithium-ion battery anodes	89
MIL-100(Fe)/Fe-Cu MOF	1073 F g^−1^	1000 mA g^−1^	Not reported	1000 cycles	Electrochemical synthesis	Supercapacitor	90
MIL-100(Fe)	83.9 mg g^−1^	N/A	Not reported	4 cycles	Hydrothermal		91
MIL-100(Fe)/GO	505 mAh g^−1^	N/A	Good columbic efficiency	2000 cycles	Solvothermal	Lithium-ion battery anode	92
MIL-100(Fe)-derived FeS/MoS_2_	325 mAh g^−1^	0.1–5.0 A g^−1^	Not reported	200 cycles	Hydrothermal	Sodium-ion batteries	93

**Table 4:** MIL-100(Fe)-based materials reported for wastewater dye remediation: a comparative overview

Material	Pollutant	Reaction time	Removal (%)	References
Mn–Co/MIL-100(Fe)	Malachite green	180 min	95%	Present study
MIL-100(Fe)	Reactive red 198	120 min	98%	94
	Tetracycline	180 min	80%	
MIL-100(Fe)@PAN	Methyl orange	60	99%	41
		H_2_O_2_		
	Rhodamine B	240	80%	
MIL-100(Fe)/GO	Methylene blue	210	95%	95
B-TiO_2_/MIL-100(Fe)	Methylene blue	60	91.12%	96
MIL-100(Fe)@SBA-15	Methylene blue		38 mg g^−1^	97
	Rhodamine B		32 mg g^−1^	
	Methyl orange		30 mg g^−1^	
	Alizarin yellow R		41 mg g^−1^	
In_2_S_3_/MIL-100(Fe)	Tetracycline	90 min	70%	98
MIL-100(Fe)	Metoprolol	300 min	10%	99
	Clofibric acid	300 min	60%	
	*N*,*N*-Diethyl-*m*-toluamide	300 min	7%	
	Ibuprofen	30 min	95%	
MIL-100 (Fe)	Methylene blue	180 min	98%	100
	Methyl orange			
MIL-100(Fe)-NH_2_(20	Rhodamine B	80 min	99.5%	101
Fe_3_O_4_@MIL-100(Fe)	Methyl orange	150 min	95.51	102
TiO_2_/MIL-100(Fe)	Methylene blue	120 min	87%	103
MIL-100(Fe)	Tartrazine	60	88%	104
BiVO_4_/MIL-100(Fe)	Basic red 46	120	88%	105
2.5-Carbon nanodots/MIL-100(Fe)	Tetracycline	30 min	82%	106
Mn_2_O_3_/MIL-100(Fe)	Rhodamine B	30 min	95.91	107

## 4 Conclusion

A modified solvothermal synthesis was employed to prepare a Mn–Co-integrated MIL-100(Fe) composite, which was successfully evaluated as a dual-function electrode material for supercapacitor applications and adsorbent for dye-contaminated wastewater remediation. The formation of the composite and the hetero-interface between the nanoparticles and MOF matrix were confirmed by FTIR, XRD, SEM, and EDX analyses, while structural characterization confirmed preservation of the MIL-100(Fe) framework following incorporation of the Mn–Co bimetallic species. BET analysis confirmed a microporous framework capable of supporting electrolyte-ion transport, and UV-vis analysis revealed an intermediate band gap of 1.85 eV, suggesting electronic interaction between the Mn–Co species and the MIL-100(Fe) framework.

Electrochemical studies performed in 1 M KOH electrolyte, conducted for both pristine MIL-100(Fe) and the Mn–Co/MIL-100(Fe) nanocomposite under identical conditions, confirmed pseudocapacitive charge-storage behavior in both electrodes. Power-law and Dunn's-method analyses revealed a marked shift in charge-storage mechanism upon Mn–Co incorporation, from a predominantly diffusion-controlled process in pristine MIL-100(Fe) (*b* = 0.45) to a predominantly surface-controlled, capacitive-dominated process in the Mn–Co/MIL-100(Fe) nanocomposite (*b* = 0.99), with the capacitive contribution reaching up to ∼84% at higher scan rates. This enhancement was directly reflected in the GCD-derived specific capacitance, with the Mn–Co/MIL-100(Fe) electrode delivering 167.9 F g^−1^ at 0.8 A g^−1^ approximately 2.5-fold higher than pristine MIL-100(Fe) (66.2 F g^−1^) under identical measurement conditions confirming that Mn–Co incorporation substantially enhances the density and accessibility of faradaic redox-active sites within the MIL-100(Fe) framework. Cycling stability evaluation over 52 charge–discharge cycles showed no capacitance fading, with the specific capacitance and coulombic efficiency both increasing progressively (to ∼170 F g^−1^ and ∼65.6%, respectively), indicative of an electrochemical activation process rather than electrode degradation. EIS analysis further supported these findings, showing a moderate charge-transfer resistance (*R*_ct_ = 31.87 Ω) consistent with efficient ion diffusion within the porous framework. Despite the need for further optimization with regard to conductivity and capacitance, the present work provides important insights into the design of bimetallic metal–organic framework-based electrode materials for next-generation energy-storage applications. Beyond its pseudocapacitive performance, the Mn–Co/MIL-100(Fe) nanocomposite exhibited excellent removal efficiency (∼95%) toward MG dye, highlighting the multifunctional potential of Mn–Co bimetallic incorporation in simultaneously enhancing MIL-100(Fe)'s energy storage and environmental remediation capabilities, and positioning it as a promising material for integrated energy and water-treatment applications. Future work will focus on evaluating the nanocomposite's adsorption selectivity toward a broader range of cationic dyes, including competitive/binary-dye studies, to clarify whether this performance is specific to MG or reflects a more generalizable capability, further establishing its applicability for complex, multi-component dye-contaminated wastewater.

## Author contributions

Ayesha Bibi: writing original draft, investigation. Arfaa Sajid: supervision, writing – review & editing. Ajwa Jabeen: methodology. Muhammad Hassan: data curation. Faiza Imtiaz: formal analysis. Awais Ahmad: project administration. Muhammad Iftikhar Khan: formal analysis. Qaisar Manzoor: writing – review & editing.

## Conflicts of interest

The authors declare that they have no known competing financial interests or personal relationships that could have appeared to influence the work reported in this paper.

## Data Availability

Data will be made available on request.
